# Induction of iPSC-derived *Prg4*-positive cells with characteristics of superficial zone chondrocytes and fibroblast-like synovial cells

**DOI:** 10.1186/s12860-022-00431-8

**Published:** 2022-07-23

**Authors:** Takashi Satake, Shingo Komura, Hitomi Aoki, Akihiro Hirakawa, Yuuki Imai, Haruhiko Akiyama

**Affiliations:** 1 Department of Orthopaedic Surgery, Gifu University Graduate School of Medicine, Gifu, Japan; 2 Department of Tissue and Organ Development, Advanced Medical Science, Gifu University Graduate School of Medicine, RegenerationGifu, Japan; 3 Division of Integrative Pathophysiology, Proteo-Science Center, Graduate School of Medicine, Ehime University, Matsuyama, Japan

**Keywords:** iPSCs, Prg4, Superficial zone chondrocytes, Fibroblast-like synovial cells

## Abstract

**Background:**

Lubricin, a proteoglycan encoded by the *PRG4* gene, is synthesised by superficial zone (SFZ) chondrocytes and synovial cells. It reduces friction between joints and allows smooth sliding of tendons. Although lubricin has been shown to be effective against osteoarthritis and synovitis in animals, its clinical application remains untested. In this study, we aimed to induce lubricin-expressing cells from pluripotent stem cells (iPSCs) and applied them locally via cell transplantation.

**Methods:**

To generate iPSCs, *OCT3/4*, *SOX2*, *KLF4*, and *L-MYC* were transduced into fibroblasts derived from *Prg4-mRFP1* transgenic mice. We established a protocol for the differentiation of iPSC-derived *Prg4-mRFP1*-positive cells and characterised their mRNA expression profile. Finally, we injected *Prg4-mRFP1*-positive cells into the paratenon, surrounding the Achilles tendons and knee joints of severe combined immunodeficient mice and assessed lubricin expression.

**Result:**

Wnt3a, activin A, TGF-β1, and bFGF were applied to induce the differentiation of iPSC-derived *Prg4-mRFP1-*positive cells. Markers related to SFZ chondrocytes and fibroblast-like synovial cells (FLSs) were expressed during differentiation. RNA-sequencing indicated that iPSC-derived *Prg4-mRFP1*-positive cells manifested expression profiles typical of SFZ chondrocytes and FLSs. Transplanted iPSC-derived *Prg4-mRFP1-*positive cells survived around the Achilles tendons and in knee joints.

**Conclusions:**

The present study describes a protocol for the differentiation of iPSC-derived *Prg4*-positive cells with characteristics of SFZ chondrocytes and FLSs. Transplantation of lubricin-expressing cells offers promise as a therapy against arthritis and synovitis.

## Background

Lubricin is a high-molecular weight proteoglycan encoded by the *PRG4* gene. It is expressed in superficial zone (SFZ) chondrocytes of articular cartilage, synovial cells, tenocytes, and the tendon sheath [1–4]. It diminishes the friction between joints and allows for smooth sliding of tendons [5–9]. Osteoarthritis causes increased chondrocyte destruction, joint friction, and synovial inflammation. When articular cartilage is destroyed in a localised superficial area, chondrocytes are replaced by fibrous cartilage tissue. When the same happens in a large and deep area, chondrocytes do not reproduce and the subchondral bone becomes exposed. Tenosynovitis is a painful condition, which causes increased synovial inflammation and adhesion of the tendon. Subsequently, prolonged tenosynovitis causes scarring of the tendon, which results in loss of its smooth sliding.

In the early stage of osteoarthritis, with only synovial inflammation, symptoms can be alleviated via intra-articular injection of hyaluronic acid or synovectomy. Similarly, mild tenosynovitis can be alleviated via injection of steroids in the tendon sheath, while in the case of deep cartilage defect in a limited area, autologous cartilage transplantation can be performed [10, 11]. An alternative therapy is cartilage tissue regeneration from mesenchymal stem cells (MSCs), embryonic stem cells (ESCs), and induced pluripotent stem cells (iPSCs) [12–18]. Owing to their pluripotency and versatility, iPSCs have been applied in regenerative medicine for several tissue and organs, including cartilage and tendon [19–22].

The therapeutic effect of lubricin administration has also garnered increasing interest. Intra-articular injections of partial recombinant human lubricin (rhPRG4) prevented cartilage degeneration in a rat model of osteoarthritis [6], and full-length rhPRG4 mitigated cartilage damage following destabilisation of the medial meniscus in a minipig model [23]. In addition to lubricating joints and tendons, rhPRG4 exhibited anti-inflammatory effect by inhibiting cytokine (interleukin-1β and tumour necrosis factor-α) secretion from osteoarthritis fibroblasts and cytokine-induced proliferation of synovial cells [24, 25]. However, according to the experimental results, repeated injections of rhPRG4 are likely required to obtain treatment effects. Moreover, rhPRG4-based therapy has not been applied clinically. Here, we hypothesised that a therapeutic strategy based on injecting iPSC-derived lubricin-expressing cells could provide an effective treatment for osteoarthritis and tenosynovitis. In this study, we aimed to induce lubricin-expressing cells from murine iPSCs with fluorescent *Prg4* reporter system and confirm the engraftment of transplanted cells in vivo.

## Methods

### Generation of iPSCs and maintenance

First, transgenic mice expressing monomeric red fluorescent protein (mRFP1) under the control of *Prg4* promoter were generated by injection of the transgene into the pronuclei of fertilised eggs from C57BL/6 mice (Charles River). Ear tips, harvested from *Prg4-mRFP1* mice, were minced and trypsinized for 15 min with 5% CO_2_ at 37 ℃ and cultured in standard high-glucose DMEM, containing 10% foetal bovine serum (Thermo Scientific), 50 units/mL penicillin (Wako), and 50 μg/mL streptomycin (Wako) until subconfluent. To establish iPSCs, fibroblasts derived from *Prg4-mRFP1* transgenic mice were infected with pMX-hOCT3/4, pMX-hSOX2, pMX-hKLF4, and pMX-Hu-L-MYC retroviral vectors (Addgene) [26]. The established iPSC clones were maintained on mitomycin C (Wako)-treated mouse embryonic fibroblasts in ESC medium consisting of KnockOut DMEM (Gibco), 15% foetal bovine serum, 2 mM L-glutamine (Wako), 1% non-essential amino acids (Wako), supplemented with 1000 units/mL human recombinant leukaemia inhibitory factor (LIF; Wako), 0.1 mM 2-mercaptoethanol (Gibco), 50 μg/mL L-ascorbic acid (Sigma-Aldrich), 50 units/mL penicillin, and 50 μg/mL streptomycin.

### Maintenance of ESCs

Murine ESCs (V6.5) were maintained on mitomycin C-treated mouse embryonic fibroblasts in ESC medium (KnockOut DMEM containing 15% foetal bovine serum, 2 mM L-glutamine, and 1% non-essential amino acids), supplemented with 1000 units/mL LIF, 0.1 mM 2-mercaptoethanol, 50 units/mL penicillin, and 50 μg/mL streptomycin.

### Teratoma formation and histological analysis

We injected 3 × 10^6^ iPSCs in 200 μl of phosphate-buffered saline into the subcutaneous tissue of severe combined immunodeficient (SCID) mice (*n* = 4) (Charles River). Three weeks after injection, the mice were sacrificed and teratomas were dissected, fixed in 4% paraformaldehyde overnight, and embedded in paraffin. Semi-serial sections were stained with hematoxylin and eosin (H&E).

### Induction of iPSC-derived *Prg4*-positive cells

Before starting the induction of *Prg4*-positive cells, iPSCs were cultured for 4 days in iPSC maintenance medium in a humidified atmosphere with 5% CO_2_ at 37 ℃, until they became subconfluent. Initially, iPSCs were differentiated as embryoid bodies on 96-well plates (Nunclon Sphera; Thermo Scientific) in standard high-glucose DMEM containing 10% foetal bovine serum, penicillin, and streptomycin for 2 days. On day 2, the medium was changed to standard medium containing 9 ng/mL activin A (Peprotech) and 25 ng/mL Wnt3a (R&D) for mesoderm differentiation. On day 3, the medium was changed to standard medium containing 10 ng/mL basic fibroblast growth factor (bFGF; Wako), and the cells were cultured continuously for 2 days. On day 5, embryoid bodies were dissociated and reseeded as a monolayer on collagen-coated 6-well plates, containing standard medium supplemented with 1% insulin-transferring-selenium (ITS; Gibco), 10 ng/mL transforming growth factor beta 1 (TGF-β1; Cell Signaling), and 10 ng/mL bFGF. Medium was changed every other day and differentiated cells were cultured until day 21.

### Fluorescence-assisted cell sorting analysis

*Prg4-mRFP1*-positive cells were selected by fluorescence-activated cell sorting (FACS) on a BD FACS Aria flow cytometer (BD Biosciences). We used ESCs-derived differentiated cells as a negative control. Data were analysed with DIVA software (BD Biosciences), which identified iPSC-derived *Prg4-mRFP1*-positive cells as having a higher PE-Texas Red signal than negative control ESC-derived cells.

### Quantitative real-time PCR

Total RNA was isolated with the RNeasy Plus Mini Kit (Qiagen) and reverse-transcribed into cDNA using the High-Capacity cDNA Reverse Transcription Kit (Applied Biosystems) according to the manufacturer’s instructions. Quantitative real-time PCR (qRT-PCR) was performed using TB Green Premix Ex Taq II (TaKaRa). The expression of target genes was normalised to that of reference genes (*Actb* and *Gapdh*) and is presented as the mean ± standard deviation based on three technical replicates per n, and *n* = 3 independent experiments. Primers used for qRT-PCR are listed in Table 1.

**Table 1:** Primer sequences

Primer sequence	Forward (5’-3’)	Reverse (5’-3’)
Actb	GCTACAGCTTCACCACCACA	CTTCTGCATCCTGTCAGCAA
Nanog	TTCCTGGTCCCCACAGTTTG	CTGGGCCTGAGAGAACACAG
Oct3/4	TCCCATGCATTCAAACTGAG	CCACCCCTGTTGTGCTTTTA
Esrrb	CAAGAGAACCATTCAAGGCAACA	CATCCCCACTTTGAGGCATTT
Rex1	TTGGAGGAAGTGGAGCAAAACC	TTCTCTTGCTTCGTCCCCTTTG
Gapdh	TGACCTCAACTACATGGTCTACA	CCGTGAGTGGAGTCATACTGG
Prg4	AGCCAATGAAGAAGTGCACAGGGA	AGGTGTGTGTCTGGAAAGGTCCAA
TenascinC	TGGGATTGGTTCTGCTGTCAA	CATTTCTTCCGTGGATGCCTTCAC
Erg	CCAGCGTCCTCAGTTAGATCCTTACCA	TCATGTTGGGCTTGCTCTTCCTCTC
Runx1	AACCCTCAGCCTCAGAGTCA	GCGATGGATCCCAGGTACT
Wnt16	GCCACTACCACTTCCACCC	GAGCCACCATTCTGCAAGG
Wnt9a	GGTGGGCAAGCACCTAAAAC	GTACAAGCTCTGGTGTTCGGG
Cadherin11	CAATATCGTTGATGGAGACGGC	ACATTGGCGGCCTCTATCTT
Col4a	CGCCTGGTACAAAAACCTCCA	CCGTGATAAAGTGCGTGCCA
Cd55	AATGCGAGGGGAAAGTGAC	TGAGGGGGTTCCTGTACTTG
Vcam1	GGAGACCTGTCACTGTCAACTG	TCCATTTCACCACTGTGTAACC
Icam1	AGACACAAGCAAGAAGACCACA	TGACCAGTAGAGAAACCCTCG
Cd248	GCCAGCAGATGTGTGTCAA	GTAGGTGCCAGCCATAGGAT

### Injection of iPSC-derived *Prg4-mRFP1*-positive cells in the paratenon surrounding the Achilles tendon and knee joint of SCID mice

We injected iPSC-derived *Prg4-mRFP1*-positive cells at 1 × 10^4^ cells/10 μl in phosphate-buffered saline in the paratenon surrounding the Achilles tendons and knee joints of SCID mice (*n* = 10) (Charles River). Three days after injection, the mice were sacrificed, and the Achilles tendons and knee joints were dissected and fixed in 4% paraformaldehyde overnight.

### Histological analysis

All tissues were fixed in 4% paraformaldehyde overnight at 4 ℃ and embedded in paraffin. The knee joint tissues of *Prg4-mRFP1* transgenic mice and samples of SCID mice, which were injected with iPSC-derived *Prg4-mRFP1*-positive cells, were fixed in 4% paraformaldehyde overnight at 4 ℃ and then decalcified in EDTA (G-Chelate Mild; NIPPON Genetics) for 2 weeks at 4 ℃. Semi-serial sections were stained with H&E. For immunohistochemistry, sections were stained with a rat monoclonal anti-RFP antibody (1:200, 5f8; Chromotek), anti-rat Histofine simple stain mouse MAX PO (414,311; Nichirei Bioscience), and DAB substrate (K3467; DAKO). For immunofluorescence, sections were stained with an Alexa 594-conjugated anti-RFP antibody (1:2000, 150,160; Abcam). Cell nuclei were stained with DAPI (1:500; Cell Signaling Technology).

### RNA-sequencing analysis

To determine their expression profile, iPSC-derived *Prg4-mRFP1*-positive cells, sorted by FACS (#2–9), were analysed on a next-generation sequencer. A heatmap summarising the genes related to the differentiation process was generated based on markers related to myogenesis, mesoderm, macrophage-like synovial cells (MLSs), pluripotency, chondrogenesis, joint interzone, fibroblast-like synovial cells (FLSs), and SFZ cells. The obtained profiles were compared to ESC (SRS1026767 from the SRA database), mesoderm (ERR2179979 from SRA), chondrocyte (GSE92641 from the GEO database), FLSs (GSE142607 from GEO), MLSs (GSE142607 from GEO), and muscle (GSE152756 from GEO) RNA-sequencing datasets.

### Statistical analysis

Statistical analysis was performed using GraphPad Prism 5 software. One-way ANOVA with Turkey’s post-test was used for statistical analysis. A *P*-value < 0.05 was considered statistically significant. Data are presented as the mean ± standard deviation based on three independent experiments.

## Results

### Generation of *Prg4-mRFP1* transgenic mice and iPSCs harbouring the *Prg4-mRFP1* reporter system

**Fig. 1:**
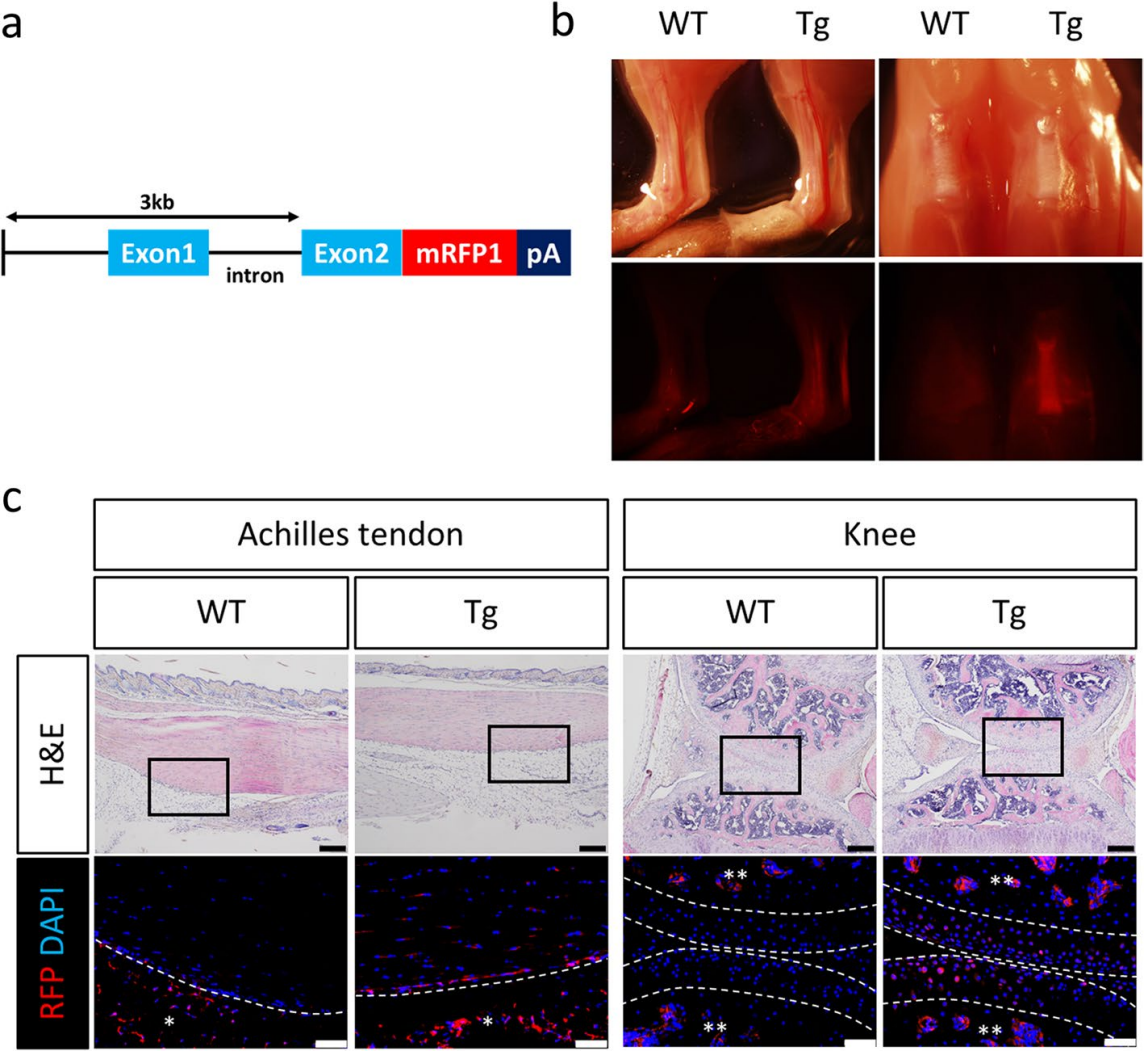
Generation of *Prg4-mRFP1* transgenic mice. **a** Schematic representation of the *Prg4-mRFP1* expression system. The poly-adenylation signal (pA), monomeric red fluorescent protein 1 (mRFP1). **b** Lateral view of the Achilles tendons and frontal view of a knee joint in wild-type (WT) and *Prg4-mRFP1* transgenic (Tg) mice. Upper panels correspond to bright-field images, and lower panels show the red fluorescence from mRFP1. **c** Histological analysis of the Achilles tendon and knee joint in WT and Tg mice. Semi-serial sections were stained with hematoxylin and eosin (H&E), anti-RFP antibody, and DAPI (for cell nuclei). Immunohistochemistry images are magnified from the squares denoted in each H&E image. Dashed white lines show the border of the tendons and articular surfaces. Scale bars: 200 μm (H&E) and 50 μm (immunohistochemistry). Background red staining of intercellular space and red blood cells by single and double asterisks, respectively

**Fig. 2:**
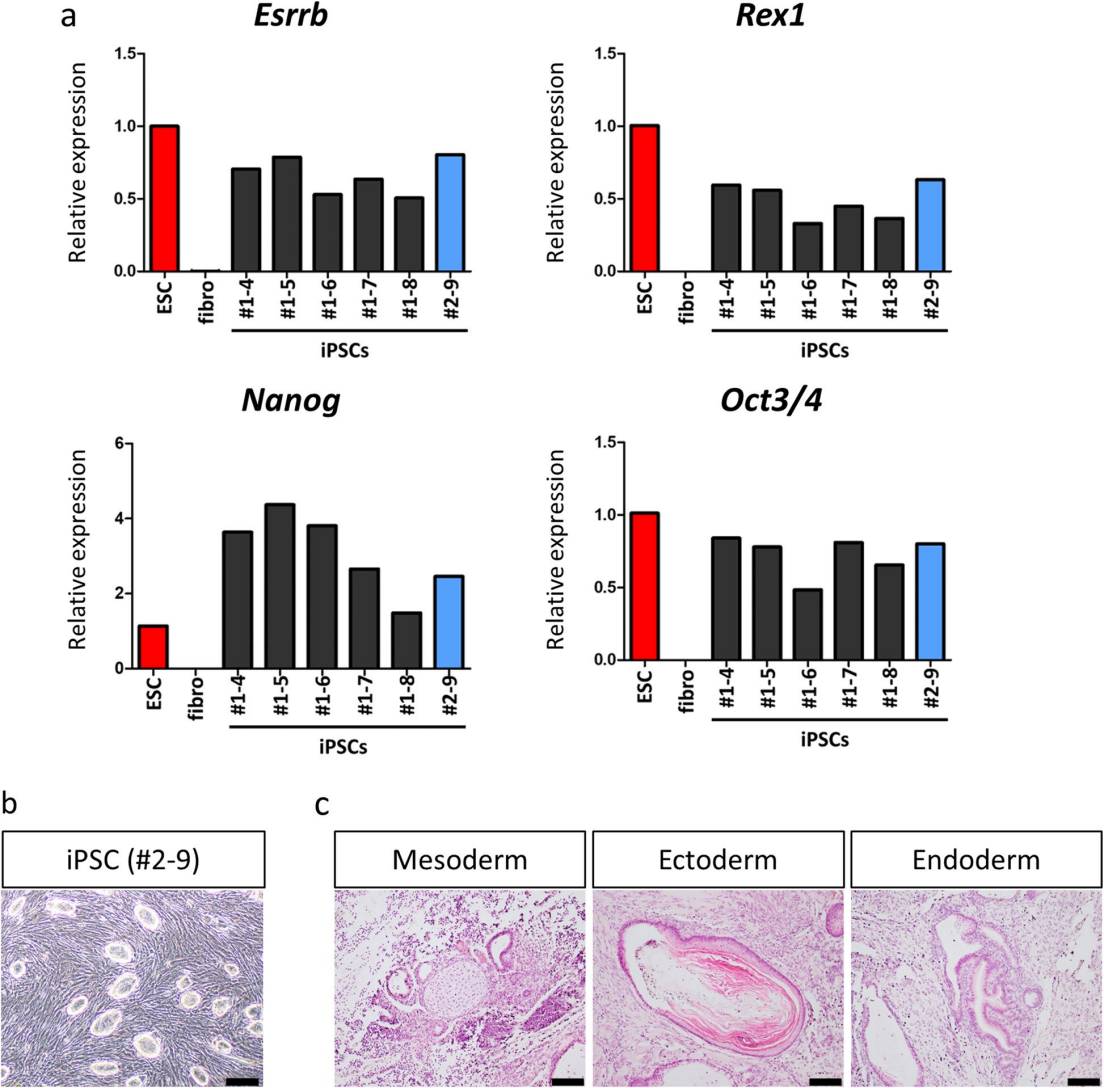
Establishment of iPSCs from *Prg4-mRFP1* transgenic mice. **a** Quantitative RT-PCR analysis of pluripotency-related genes in embryonic stem cells (ESCs), ear tip fibroblasts (fibro), and six iPSC clones. The expression level of ESCs was set to 1. Data are presented as the mean of three technical replicates. **b** Bright-field image of iPSCs (#2–9). Scale bar: 200 μm. **c** Teratomas derived from iPSCs (#2–9) comprising ectodermal, mesodermal, and endodermal tissue. Scale bars: 100 μm

To create the *Prg4-mRFP1* transgene, we inserted the *mRFP1* expression cassette, which contained a Kozak sequence and poly-adenylation signal, 3 kb downstream of the *Prg4* promoter (Fig. 1a). The transgenes were injected in the pronuclei of fertilised eggs from C57BL/6 mice to generate *Prg4-mRFP1* transgenic mice. To verify the successful construction of transgenic mice, mRFP1 fluorescence was detected around the Achilles and patellar tendons (Fig. 1b); whereas immunohistochemistry showed mRFP1 expression in tenocytes, tendon synovium, and SFZ chondrocytes of the articular cartilage (Fig. 1c). These results suggested that the *Prg4-mRFP1* expression system was active in vivo and could be used to monitor the production of lubricin. Next, we generated iPSCs harbouring the *Prg4-mRFP1* reporter system. iPSC-like cells were established from fibroblasts of *Prg4-mRFP1* transgenic mice by retroviral transduction with *OCT3/4, SOX2, KLF4,* and *L-MYC*. Six iPSC-like clones were selected, and qRT-PCR revealed a comparable expression of pluripotency-related genes between iPSCs and ESCs (Fig. 2a). Clone iPSC (#2–9) was the most similar to ESCs (Fig. 2b) and was chosen for transplantation into the subcutaneous tissue of SCID mice. Three weeks after transplantation, teratomas comprising ectodermal, mesodermal, and endodermal tissues could be observed (Fig. 2c), confirming the pluripotency of the established iPSCs.

### Differentiation of iPSC-derived *Prg4*-positive cells

**Fig. 3:**
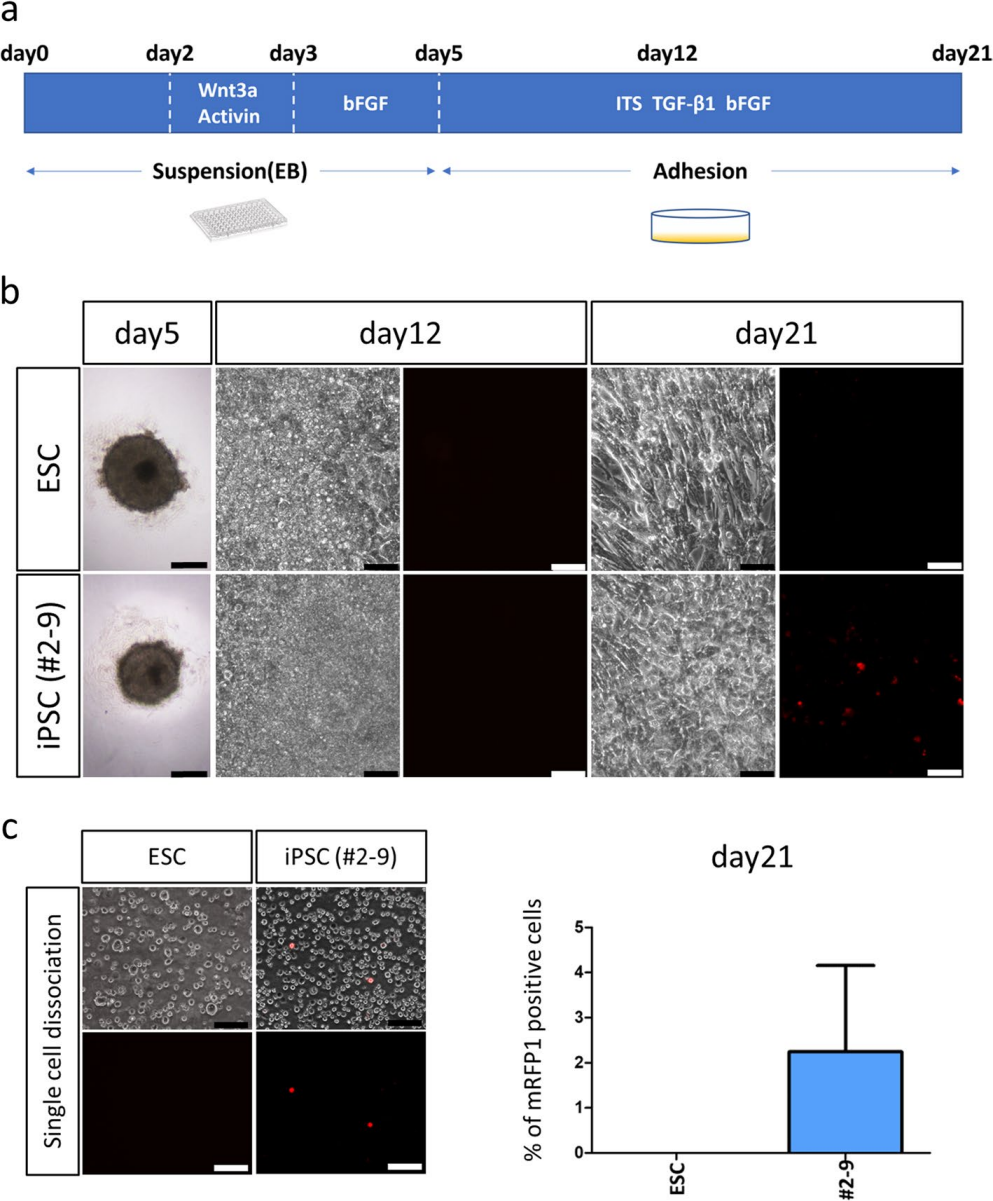
Establishment of a protocol for the differentiation of iPSC-derived *Prg4-mRFP1*-positive cells. **a** Schematic representation of the protocol used for the differentiation of iPSC-derived *Prg4-mRFP1-*positive cells. Embryoid bodies (EB) were cultured in suspension for the first 5 days and adhered to culture plates after that. Different differentiation factors were supplied. **b** Representative bright-field and fluorescence images of embryonic stem cells (ESCs) and iPSCs (#2–9) undergoing differentiation on days 5, 12, and 21. Scale bars: 200 μm (day 5) and 50 μm (days 12 and 21). **c** Quantification of iPSC-derived *Prg4-mRFP1*-positive cells on day 21. *mRFP1*-positive cells were counted after single cell dissociation (left figures). Their average on day 21 was 2.2% (maximum 7.0%). The mean of 13 independent experiments is shown. Scale bars: 50 μm

Next, we established a protocol for the differentiation of iPSC-derived *Prg4*-positive cells (Fig. 3a). First, iPSCs (#2–9) were differentiated as embryoid bodies for 2 days. On day 2, activin A and Wnt3a were added to promote differentiation of the primitive streak [24, 27–29], followed by that of the paraxial mesoderm and somites [30, 31]. Between day 3 and day 5, embryoid bodies were cultured in the presence of bFGF to promote cell proliferation and differentiation of the sclerotome. On day 5, embryoid bodies were dissociated and reseeded as a monolayer supplemented with ITS, TGF-β1, and bFGF. TGF-β1 and bFGF have been shown to promote the differentiation of SFZ chondrocytes and synovial cells from the joint interzone [30, 32–37]. On day 21, mRFP1 fluorescence was detected in iPSC-derived differentiated cells. The average of *Prg4-mRFP1* positive cell-ratio was 2.2% (maximum 7.0%), confirming the successful generation of *Prg4*-positive cells from iPSCs (Fig. 3b and c).

### Gene expression analysis of iPSC-derived *Prg4-mRFP1*-positive cells by qRT-PCR

**Fig. 4:**
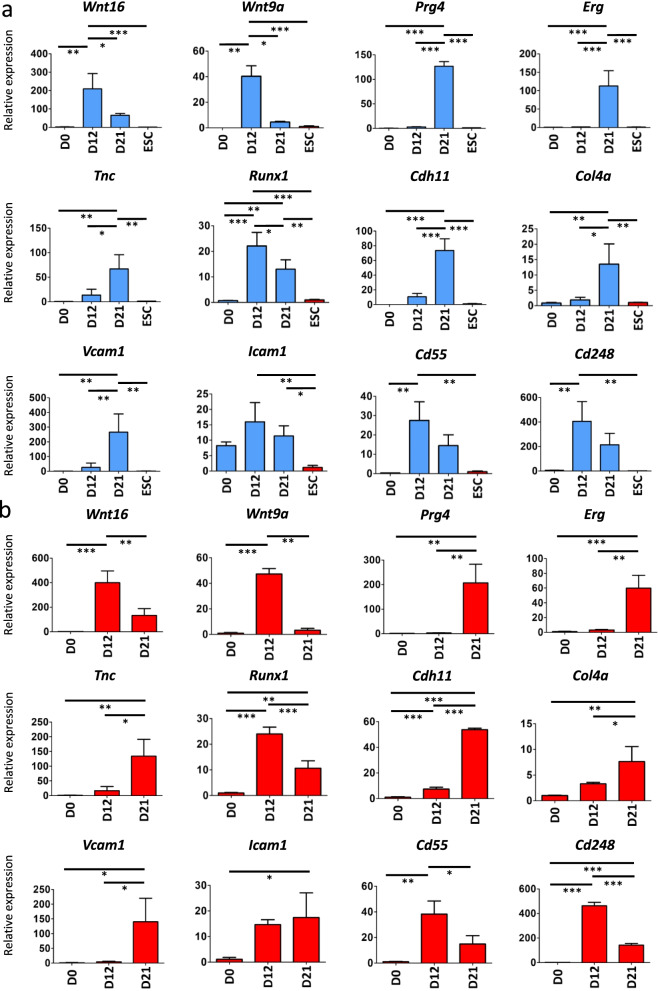
Analysis of mRNA expression in iPSC and ESC-derived differentiated cells over time. **a** mRNA expression in iPSC-derived differentiated cells regarding the genes related to the joint interzone (*Wnt16* and *Wnt9a*), superficial zone (*Prg4*, *Erg*, *Tnc* and *Runx1*) and fibroblast-like synovial cells (*Cdh11*, *Col4a1*, *Vcam1*, *Icam1*, *Cd55* and *Cd248*). Data are expressed as the mean ± standard deviation of three technical replicates per n and *n* = 3 biological replicates. *P < 0.05; **P < 0.01; and ***P < 0.001. D0, day 0; D12, day 12; and D21, day 21. Embryonic stem cells (ESCs) were used for comparison. **b** mRNA expression in ESC-derived differentiated cells regarding the genes related to the joint interzone, superficial zone, and fibroblast-like synovial cells. Data are expressed as the mean ± standard deviation of three technical replicates per n and *n* = 3 biological replicates. *P < 0.05; **P < 0.01; and ***P < 0.001. D0, day 0; D12, day 12; and D21, day 21

To investigate the differentiation state of iPSC-derived cells generated with our protocol, we analysed the expression of target mRNAs by qRT-PCR over time. *Prg4* is expressed in SFZ chondrocytes and synovial cells [1–4]. The latter are classified mainly into FLSs and MLSs [38, 39]. FLSs, which are of mesenchymal origin, secrete lubricin and play a critical role in normal joint homeostasis [39, 40]. Both SFZ chondrocytes and FLSs differentiate from the joint interzone during the development of synovial joints [41–43]. Therefore, we confirmed the expression of genes representative of the joint interzone (*Wnt9a* and *Wnt16*) [41–43], SFZ (*Prg4*, *Erg*, *Tnc*, and *Runx1*) [44–48], and FLSs (*Cdh11*, *Col4a*, *Vcam1*, *Icam1*, and *Cd248*) [49–55]. Expression of *Wnt9a* and *Wnt16* was significantly higher on day 12, but dropped on day 21 (Fig. 4a). Consistent with mRFP1 fluorescence on day 21, *Prg4* expression significantly increased on day 21, together with that of SFZ-related genes *Erg* and *Tnc* (Fig. 4a) and FLS-related genes *Cdh11*, *Col4a*, and *Vcam1* (Fig. 4a). Our protocol also caused the differentiation of ESCs up until day 21, which we verified by qRT-PCR. ESC-derived differentiated cells displayed a similar gene expression profile as iPSC-derived *Prg4*-positive cells (Fig. 4b). These results suggested that our differentiation protocol successfully induced *Prg4*-positive cells from pluripotent stem cells and equipped them with SFZ chondrocyte and FLS characteristics.

### RNA-sequencing analysis of FACS-sorted iPSC-derived *Prg4-mRFP1*-positive cells

**Fig. 5:**
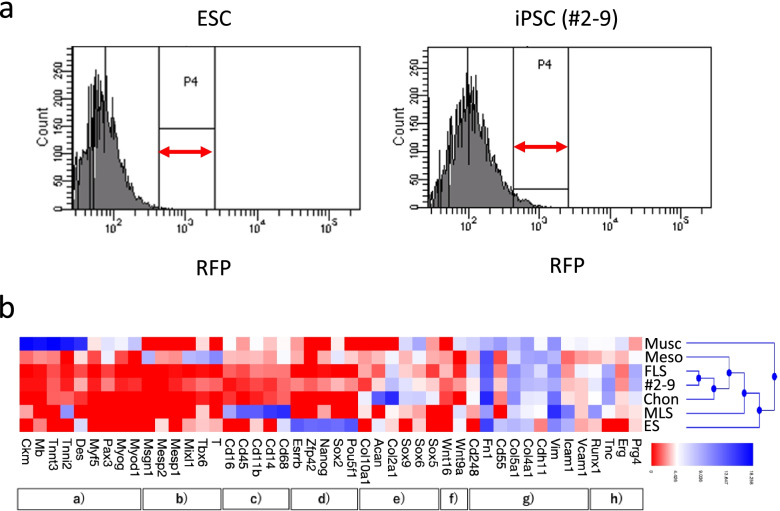
RNA-sequencing analysis of *mRFP1*-positive cells obtained by FACS. **a** Flow cytometry analysis of embryonic stem cell (ESC)-derived differentiated cells (control) and iPSC-derived *mRFP1*-positive cells (#2–9) on day 21. P4 gate of negative control was set to < 0.1%. Positive cell ratio of iPSC-derived *mRFP1*-positive cells was average 4.9% (maximum 9.3%) (*n* = 16 independent experiment). **b** Heatmap showing clustering analysis of RNA-sequencing data obtained from muscle (Musc), mesoderm (Meso), fibroblast-like synovial cells (FLS), iPSC-derived *mRFP1*-positive cells (#2–9), chondrocytes (Chon), macrophage-like synovial cells (MLS), and ESCs (ES). Myogenic (a) mesoderm (b), MLS (c), pluripotency (d), chondrogenic (e), interzone (f), FLS (g), and superficial zone (h) markers were assessed

Having demonstrated the successful induction of *Prg4-mRFP1-*positive cells by our differentiation protocol, we proceeded with their further characterisation. Based on FACS, *Prg4-mRFP1*-positive cells constituted 4.9% (maximum 9.3%) of the cells differentiated from the iPSC (#2–9) clone on an average (Fig. 5a). RNA-sequencing of mRFP1-positive cells obtained by FACS revealed that iPSC-derived *Prg4-mRFP1*-positive cells lost the properties of pluripotent stem cells, mesoderm, and joint interzone; however, they showed the properties of SFZ chondrocytes and FLSs, but not those of deep zone chondrocytes (Fig. 5b).

### Injection of iPSC-derived *Prg4-mRFP1*-positive cells in the paratenon around the Achilles tendons and knee joints of SCID mice

**Fig. 6:**
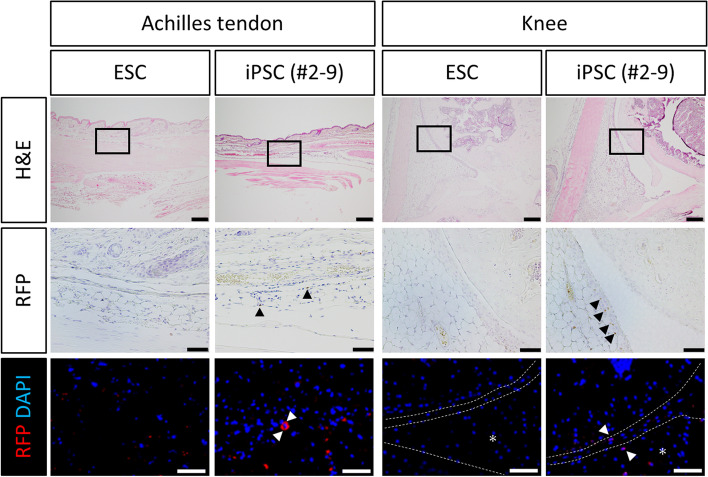
Transplantation of iPSC-derived *Prg4-mRFP1*-positive cells into SCID mice. Histological analysis of Achilles tendons and knee joints of SCID mice injected with either embryonic stem cell (ESC)-derived cells (control) or FACS-selected iPSC-derived *Prg4-mRFP1*-positive cells (#2–9). Semi-serial sections were stained with hematoxylin and eosin (H&E) and anti-RFP antibody (RFP). Middle panels of immunohistochemistry images are magnified from the squares in the corresponding upper panels (H&E staining). Lower panels of RFP immunofluorescent images are magnified from cell transplanted region of Achilles tendons and knee joints. Arrows point to mRFP1-positive cells. Asterisk indicates meniscus. Scale bars: 200 μm (upper panels),50 μm (middle panels) and 25 μm (lower panel)

Finally, we injected iPSC-derived *Prg4-mRFP1*-positive cells obtained by FACS in the paratenon surrounding the Achilles tendons and knee joints of SCID mice. Immunohistochemical analysis showed that 3 days after injection, some iPSC-derived *Prg4-mRFP1*-positive cells were still present around the Achilles tendons and in the knee joint (Fig. 6). Notably, no teratomas were observed in SCID mice at 3 weeks after cell transplantation (data not shown). These results indicated that transplanted iPSC-derived *Prg4-mRFP1*-positive cells could produce lubricin in vivo for at least 3 days without the risk of tumour formation.

## Discussion

Lubricin is believed to be an effective therapeutic agent for osteoarthritis and synovitis. However, clinical application of recombinant or synthesised lubricin has not been attempted yet. In this study, we successfully developed a protocol to induce lubricin-expressing cells from pluripotent stem cells and confirmed their differentiation into *Prg4*-expressing cells that shared the characteristics of SFZ chondrocytes and FLSs from the joint interzone.

*Prg4* is expressed in SFZ chondrocytes, synovial cells, synovial fluid, tendons, and tendon sheath [3, 4]. Such localisation was confirmed in our *Prg4-mRFP1* transgenic mice. First, iPSCs were established by transducing ear tip mouse fibroblasts with all four Yamanaka factors [26]. Second, we devised a protocol for the differentiation of iPSC-derived *Prg4*-positive cells, focusing on differentiation into SFZ cells and FLSs, which formed the synovial joint rather than cartilage. To induce the primitive streak, and subsequent differentiation into paraxial mesoderm and somites, we followed previous reports on embryoid body formation in suspension or adhesion for 4–6 days [11, 12, 15]. To maximise differentiation, we chose embryoid body formation in suspension for 5 days. Based on known cytokines involved in this process [12, 14, 15], we selected Wnt3a, activin A, and bFGF to promote differentiation of the sclerotome as a precursor to the synovial joint. Although useful for induction of the primitive streak, bone morphogenetic protein (BMP) 4 was not applied in this case as it could not lead to differentiation of the paraxial mesoderm.

For chondrogenic differentiation, most studies have employed serum-free medium [13–15] or 1% foetal bovine serum [12], as well as the cytokines ITS, bFGF, BMP2, TGF-β1, TGF-β3, growth differentiation factor 5, TD-198946, and ascorbic acid. Here, we cultured iPSCs in medium containing 10% foetal bovine serum, ITS, bFGF, and TGF-β1 to differentiate *Prg4*-positive SFZ cells and FLSs of the synovial joint.

To confirm successful differentiation, we analysed mRNA expression of iPSC-derived differentiated cells over time using qRT-PCR. *Wnt9a* and *Wnt16* are important in the joint interzone, for the development of synovial joints [41–43]. *Wnt16* also supports the phenotype of SFZ progenitor cells and lubricin expression [56]. During differentiation, *Wnt9a* and *Wnt16* showed significant upregulation on day 12, followed by a drop on day 21. This expression dynamics suggested that iPSCs differentiated in the joint interzone on day 12 and advanced to the next differentiation stage thereafter.

Besides *Prg4*, other genes expressed in SFZ chondrocytes include *Erg, Tnc*, and *Runx1* [44–48]. We confirmed increased expression of *Prg4*, *Erg*, and *Tnc* on day 21, but could hardly detect them on day 12. *Cdh11*, *Col4a*, *Cd55*, *Vcam1*, *Icam1*, and *Cd248* are expressed in FLSs of synovial joints [49–55]. Here, SFZ chondrocyte markers *Prg4*, *Erg*, and *Tnc* exhibited a similar expression pattern as FLS markers *Cdh11*, *Col4a*, and *Vcam1*. These results suggest that iPSCs differentiated into *Prg4*-expressing cells via the joint interzone. Analysis of mRNA expression in ESC-derived differentiated cells produced a similar temporal pattern as in iPSCs, suggesting that our protocol was applicable to all pluripotent stem cells. RNA-sequencing confirmed that iPSC-derived *Prg4*-positive cells (clone #2–9) were more similar to SFZ chondrocytes and FLSs than to deep zone chondrocytes, mesodermal cells, myogenic cells, and MLSs, suggesting that iPSC-derived *Prg4*-positive cells shared the characteristics of both SFZ chondrocytes and FLSs in synovial joints. Indeed, SFZ chondrocytes and FLSs are representative lubricin-expressing cells, showing higher expression than deep chondrocytes, mesoderm cells, myogenic cells, and MLSs. Therefore, the results of this study are significant in that high lubricin-expressing cells with SFZ chondrocytes and FLS characters were induced.

Recent strategies in regenerative therapy favour the use of stem cells rather than autologous cartilage transplantation to replenish missing or damaged cartilage [10, 11]. Examples of such an approach include the generation of articular cartilage using human ESCs [15], generation of scaffold-free hyaline cartilage from human iPSCs [12], generation of *Col2a1-EGFP* iPSCs for monitoring chondrogenic differentiation [13], the formation of stable human articular cartilage using human MSCs [16, 17], cartilage repair using a scaffold-free construct derived from porcine synovial MSCs [18], and specification of chondrocytes and cartilage tissue from murine ESCs [14]. The present study aimed to provide lubrication for arthritic joints and tenosynovitis by transplanting *Prg4*-expressing cells rather than regenerating damaged cartilage tissue. Hence, we injected iPSC-derived *Prg4-mRFP1-*positive cells into the paratenon surrounding the Achilles tendon and knee joints of SCID mice and confirmed the cells’ survival and lubricin expression in vivo. A recent study revealed that *Prg4*-lineage cells in the joint SFZ differentiated into articular chondrocytes [57], suggesting that injection of iPSC-derived *Prg4*-expressing cells might also have the potential to regenerate articular cartilage. Major advantages of our approach are the potential to obtain a large number of Prg4-expressing cells by using iPSCs, that harbour high capacity of self-renewal. Regarding the cell source, we can use mesenchymal stem cells with *Prg4-mRFP1* reporter system and develop differentiation protocol from MSC to *Prg4*-positive cells. Differentiation efficiency of MSCs to *Prg4*-positive cells could become better because usage of MSCs can skip the process of iPSC to mesenchymal lineage differentiation. Mature *Prg4*-expressing cells could also be available, however, it may be difficult to obtain and expand a large number of *Prg4*-expressing cells in clinical practice, especially in the elderly patients.

Finally, several hurdles need to be overcome prior to the clinical application of *Prg4*-expressing cells. First, disadvantage of our approach is the insufficient induction efficiency of *Prg4-mRFP1* positive cells, with average 4.9% (maximum 9.3%). Also, using the same protocol, we previously induced tenocyte-like cells from *Scx-EGFP* iPSCs [58]. Tenocytes share the same developmental process through somite to sclerotome with chondrocytes and synovial cells. Given that *Prg4*-expression has been confirmed in tendons, iPSC-derived *Prg4*-positive cells in this study might contain tenocytes. Therefore, the differentiation protocol should be optimised to improve the induction efficiency and purity of *Prg4*-epressing cells presenting SFZ and FLS characteristics. Second, we need to prolong the survival of these cells after injection. Transplanted *Prg4-mRFP1*-positive cells were detected in the paratenon surrounding the Achilles tendons and knee joints, but only for up to 3 days. Use of scaffold materials or platelet-rich plasma might offer a physical means for improved temporal survival of iPSC-derived *Prg4*-expressing cells [59, 60]. Also, condition media containing cytokines, growth factors, and exosomes has been used for regenerative medicine and many studies have shown their efficacy [12]. Indeed, our previous study regarding Scx-EGFP expressing iPSC-derived tenocyte transplantation revealed that cell transplantation had dual role, a direct contribution for regenerative tendon and an indirect effect (paracrine effect) reducing scar formation [58]. Of note, paracrine effect was presumed to contribute more preferentially for regeneration, indicating that cytokines, growth factors, and exosomes released from induced cells have an important role in regeneration. Therefore, the condition media of *Prg4*-*mRFP1* expressing cells could become an alternative regenerative agent against osteoarthritis and synovitis, and might also prolong the survival of *Prg4-mRFP1* positive cell after transplantation. Moreover, transplantation of *Prg4*-expressing cells might be beneficial in terms of such paracrine effect other than lubricin compared to rhPRG4 administration therapy. Third, to confirm engraftment of iPSC-derived *Prg4*-expressing cells in vivo, we used SCID mice for the transplantation experiment to avoid immune rejection. However, transplantation for osteoarthritis or synovitis model could be required to confirm the treatment effect of iPSC-derived *Prg4*-expressing cells. We would need further experiments to reveal its therapeutical benefit in the future.

## Conclusions

The present study describes the generation of murine iPSCs harbouring a *Prg4-mRFP1* reporter system and the establishment of a protocol for the differentiation of pluripotent stem cells into *Prg4*-expressing cells exhibiting the characteristics of SFZ chondrocytes and FLSs. Injection of lubricin-producing cells in the joints and paratenon around the tendons could become a viable therapeutic approach for osteoarthritis and tenosynovitis.

## Data Availability

RNA-seq data of iPSC-derived *Prg4-mRFP1*-positive cells is available in the SRA database (PRJNA839189).

## Abbreviations

bFGF: Basic fibroblast growth factor
BMP: Bone morphogenetic protein
ESC: Embryonic stem cell
FLS: Fibroblast-like synovial cell
iPSC: Induced pluripotent stem cell
ITS: Insulin-transferring-selenium
LIF: Leukaemia inhibitory factor
MLS: Macrophage-like synovial cell
MSC: mesenchymal stem cell
qRT-PCR: quantitative real-time PCR
rhPRG4: recombinant human lubricin
RFP: red fluorescent protein
SFZ: superficial zone
TGF-β: transforming growth factor beta
